# *In vivo* standardization of bone ultrasonometry of the clavicle

**DOI:** 10.6061/clinics/2016(03)04

**Published:** 2016-03

**Authors:** Luiz Garcia Mandarano-Filho, Márcio Takey Bezuti, Cláudio Henrique Barbieri

**Affiliations:** Universidade de São Paulo, Faculdade de Medicina de Ribeirão Preto, Hospital das Clínicas, Ortopedia, Ribeirão Preto/SP, Brazil

**Keywords:** Bone, Clavicle, Ultrasound

## Abstract

**OBJECTIVE:**

The assessment of fracture union includes physical examination and radiographic imaging, which depend on the examiner's experience. The development of ancillary methods may avoid prolonged treatments and the improper removal of implants. Quantitative bone ultrasonometry has been studied for this purpose and will soon be included in clinical practice. The aims of the present study were to assess the feasibility of using this technique on the clavicle and to standardize its *in vivo* application.

**METHODS:**

Twenty adult volunteers, including 10 men and 10 women without medical conditions or a previous history of clavicle fracture, underwent axial quantitative ultrasonometric assessment using transducers in various positions (different distances between the transducers and different angulations relative to the clavicle).

**RESULTS:**

Similar values of wave propagation velocity were obtained in the different tested set-ups, which included distinct distances between the transducers and angular positions relative to the clavicle. There were significant differences only in the transducers positioned at 0° and at 5 or 7 cm apart.

**CONCLUSIONS:**

The use of bone ultrasonometry on the clavicle is feasible and the standardization of the technique proposed in this study (transducers placed at 45° and at 7 cm apart) will allow its future application in clinical trials to evaluate the healing process of diaphyseal fractures of the clavicle.

## INTRODUCTION

In practice, the assessment of fracture union is performed by physical examination and serial radiographic imaging; both are dependent on the individual experience and clinical judgment of the examiner 1. The development of more objective quantitative methods may aid in the diagnosis and treatment of cases of nonunion and delayed union, in the determination of fracture union completion and in the avoidance of prolonged treatments and the improper removal of implants or external fixators 2.

The literature contains many studies that have used noninvasive quantitative methods for this purpose, such as bone densitometry 3,4, vibration analysis 5, and acoustic emission 6,7.

Ultrasound methods that investigate the appearance and neovascularization of the callus have been studied for use in the assessment of fracture healing 8,9,10; however, most studies have used qualitative techniques. Quantitative ultrasound using axial transmission is the most appropriate technique for long bones 2. This technique uses transmitter and receiver transducers that are placed in direct contact with the skin on either side of the fracture site. The ultrasound waves propagate along the longitudinal axis of the bone at different speeds in the cortical bone and in the region of the forming callus when compared with the intact bone, used as a reference.

Several studies have reported the use of quantitative ultrasonometry during the process of fracture union in animal bones 11,12,13,14, including certain *in vivo* studies 15,16,17. The few studies that have been conducted in humans have focused on the tibia 18,19,20,21. The aims of the present study were to assess the feasibility of using bone ultrasonometry on the clavicle and to standardize this technique.

## MATERIALS AND METHODS

**Figure 1-:**
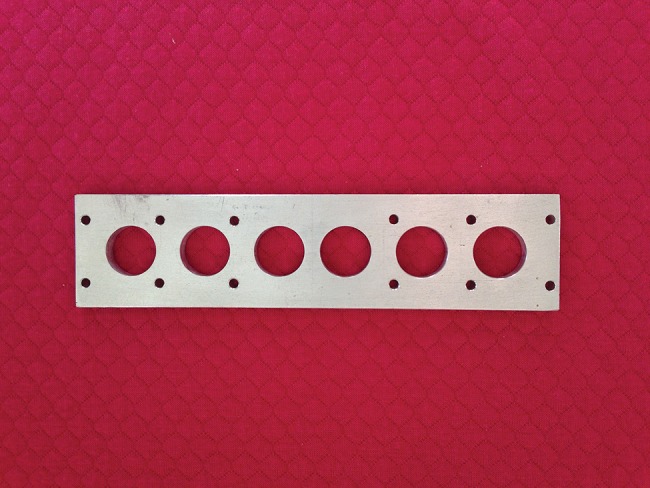
Aluminum holder made to couple the transducers (upper view).

This study was approved by the research ethics committee of the authors' institution. Twenty adult volunteers (25–57 years of age), including 10 men and 10 women without medical conditions or a previous history of fracture of the clavicle, underwent ultrasonometric assessment using transducers placed in various positions (different distances between the transducers and different angulations relative to the clavicle). An aluminum holder with holes in it was made to couple the transducers, enabling them to be positioned at varying distances from the holder's geometric center (3, 5, and 7 cm) (Figure 1).

Two transducers (one transmitter and one receiver) were used for this purpose. They consisted of plates in the shape of a disc of 15 mm in diameter and were made of PZT-5, a ceramic material with piezoelectric properties. The transducers were connected to a generator-receptor-amplifier of ultrasound pulses (Biotecnosis do Brasil Ltda., Model US01, Brazil), which was linked to an oscilloscope (Agilent Technologies Inc., Digital Storage Oscilloscope 3062A, China) to visualize the input signal. The latter was in turn connected to a microcomputer with software (Biotecnosis do Brasil Ltda., Brazil) for signal processing and calculating the ultrasound propagation velocity. The ultrasound device that was used works with a circuit that generates narrow pulses at a frequency of 1 MHz. Although the input voltage at the source transducer is adjustable, it was set to 100 V, fixing the voltage applied to the emitter transducer, with sufficient power for the pulse to be transmitted through the bone sample without being completely attenuated.

The signal received by the receiver transducer was amplified by a specific circuit with a selector switch; an amplification factor of three was established for better visualization of the waves. The oscilloscope read the wave output, and the microcomputer processed the received signals and stored the information.

During velocity calculation, it is important to identify the first arrived signal, which defines the travel time. Different references can be used to detect the input of the signal; in the present study, the reference was defined as a wave deflection of >5% from the baseline, which was automatically calculated by the computer program.

The device was calibrated using a polytetrafluorethylene cylinder with a known, constant ultrasound propagation velocity. The cylinder was placed between the transducers so that the ultrasound wave would fall onto the flat surface of the piece, and coupling gel was used between the transducers and the polytetrafluorethylene cylinder. This procedure was repeated before the assessment of each patient to ensure reproducibility of the measurements. The mean ultrasound propagation velocity in the Teflon cylinder was 1.156 m/s.

**Figure 2-:**
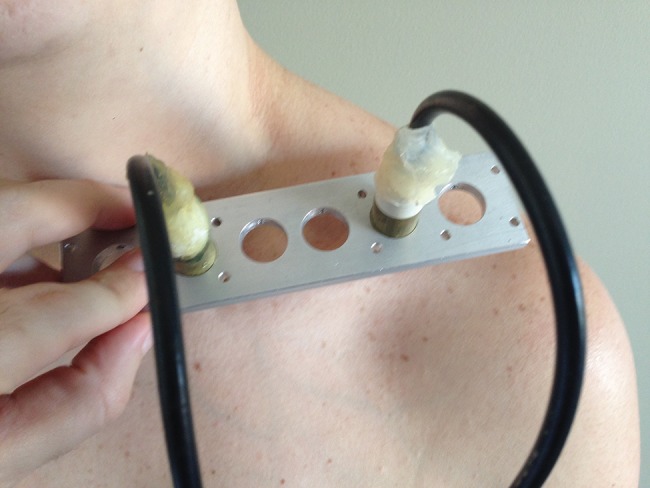
System positioned *in vivo* at a 45° angle.

**Figure 3-:**
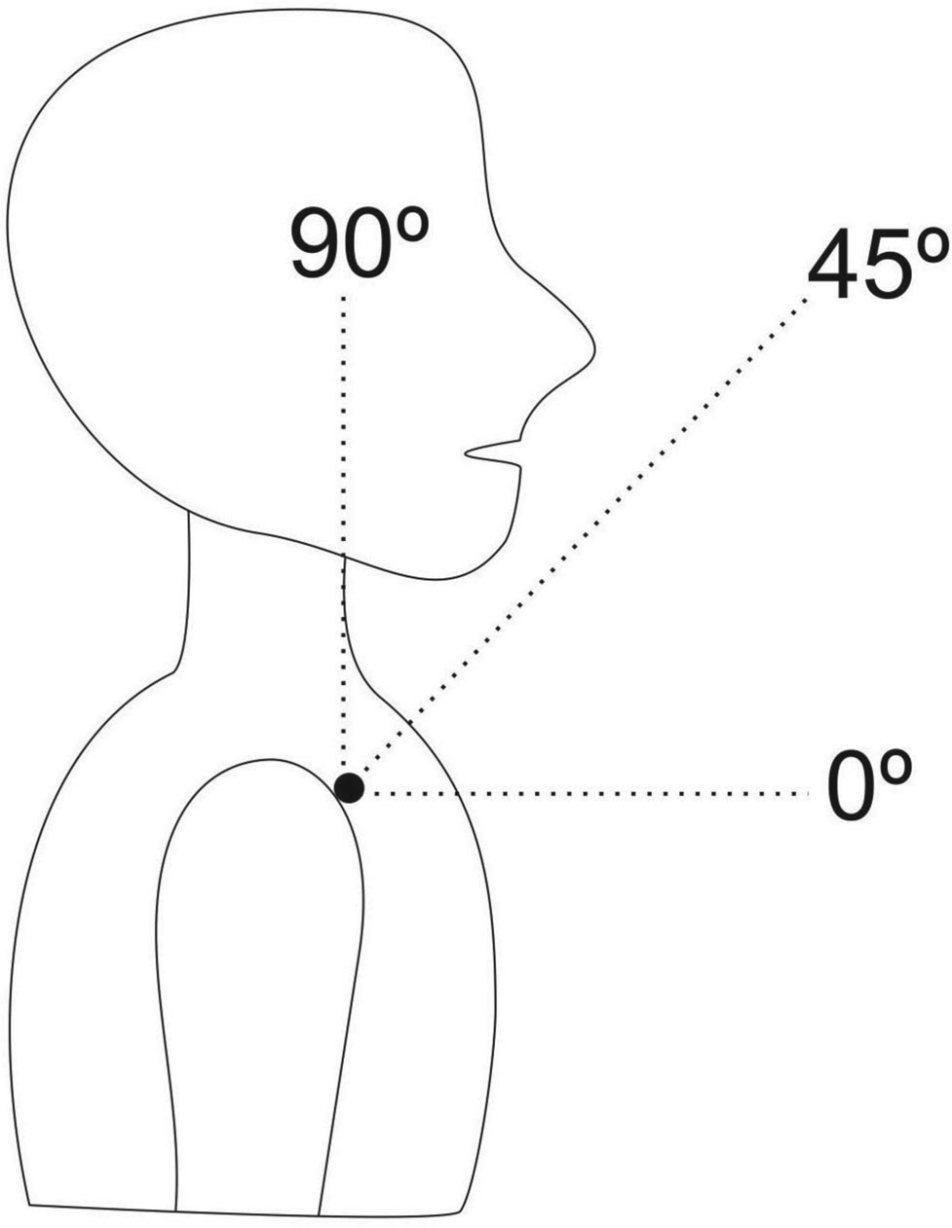
Schematic representation of the angulation of the transducers.

Bone ultrasonometry was performed on both clavicles of each volunteer, with the transducers positioned 3, 5, or 7 cm apart and at three different angles relative to the ground (0°, 45°, and 90°) (Figure 2). The angulation of the system was set up with the aid of a manual goniometer, with one arm parallel to the ground and the other arm aligned with the transducers' axis (Figure 3). Gel was placed between the transducers and the skin in all cases.

For statistical analyses, Student's *t*-tests were used, which compare two means of non-paired samples. For this test, it is necessary to first assess whether the variances of the two groups are statistically similar and whether the data follow a normal distribution. Here, this was performed using the PROC TEST procedure of SAS^®^ 9.0 software.

Furthermore, ANOVA was performed, which involves partitioning the total variance of a given response (dependent variable) into two parts: the first part relates to the regression model and the second part relates to the residuals. The greater the ratio between the first and the second parts is, the greater the evidence of a difference between the means of the groups is. This model assumes that the residuals follow a normal distribution, with a mean of 0 and constant variance. When this assumption was not fulfilled, a transformation was applied to the response variable. This procedure was performed using the PROC GLM procedure of SAS^®^ software. Orthogonal contrasts based on the *t* distribution were used for the comparisons.

Mixed-effects (random and fixed effects) linear models are used in the analysis of data when the responses of an individual are grouped and the assumption of independence between observations within one group is not adequate.

In the mixed-effects model that was used here, individuals were considered as random effects, and distances, times, and the interaction between these were considered as fixed effects. This model assumes that the residual obtained from the difference between the values predicted by the model and the observed values has a normal distribution, with a mean of 0 and constant variance. The model was adjusted using the PROC MIXED procedure of SAS^®^ 9.0 statistical software.

## RESULTS

First, a descriptive analysis of the results was performed. The mean age of the 20 volunteers was 41.3 years (25–60 years, median of 40.5, and standard deviation of 11.5). The mean wave propagation velocities were 3062.88 m/s in women and 3059.73 m/s in men, independent of the angulation and distance between the transducers, without any significant difference (*p*=0.6018).

Comparison between the sides showed mean propagation velocities of 3061.66 m/s on the right side and 3060.96 m/s on the left side, independent of the angulation and distance between the transducers, without any significant difference (*p*=0.9079).

**Figure 4-:**
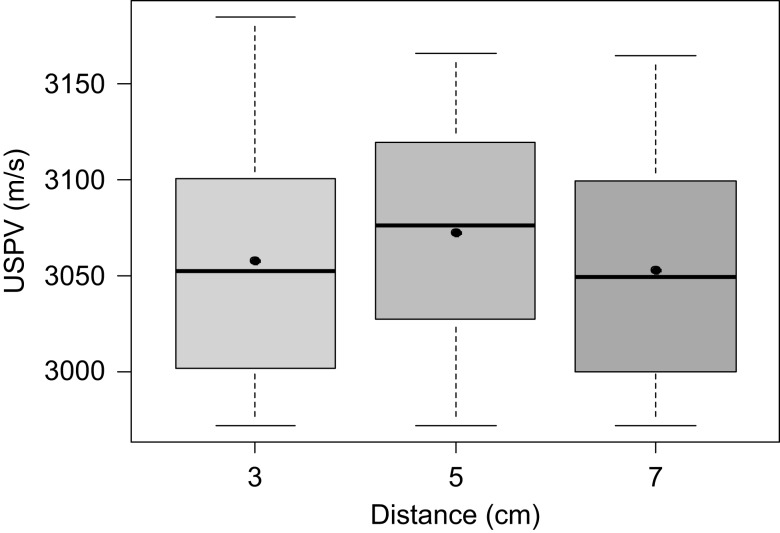
Ultrasound propagation velocity (m/s) comparison between the distances (3, 5 and 7 cm) of the transducers, regardless of their angulation.

The mean propagation velocities obtained for the 3, 5 and 7 cm distances between transducers, regardless of their angulation, were 3057.98, 3072.75 and 3053.18 m/s, respectively. There was a significant difference between the 3 and 5 cm distances (*p*=0.0448) and between the 5 and 7 cm distances (*p*=0.0080), whereas t. There was no significant difference between the 3 and 7 cm distances (*p*=0.5132). These results are shown in Figure 4.

**Figure 5-:**
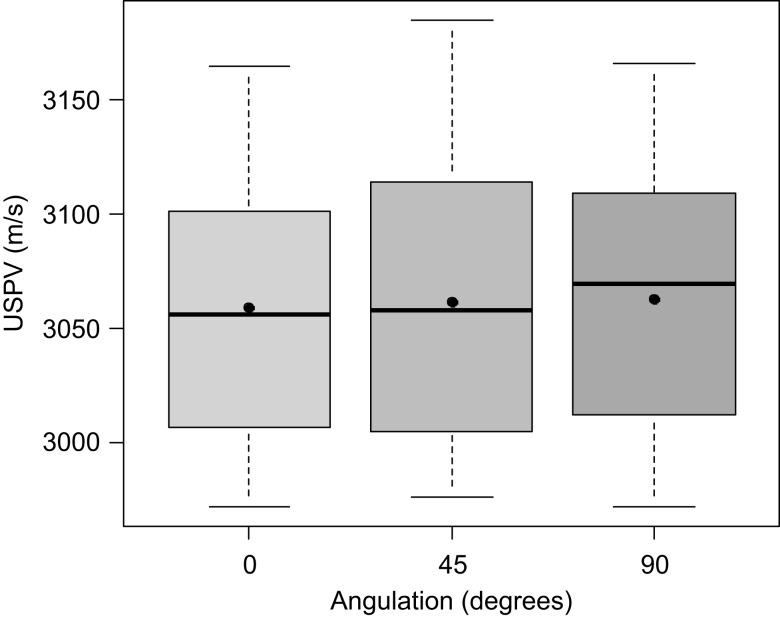
Ultrasound propagation velocity (m/s) comparison between the angulations (0°, 45° and 90°) of the transducers, regardless of the distance between them.

The mean propagation velocities obtained for the angulations of the transducers (0°, 45° and 90°), independent of the distance between them, were 3059.17, 3061.88 and 3062.88 m/s, respectively. There was no significant difference between the groups, as shown in Figure 5.

**Figure 6-:**
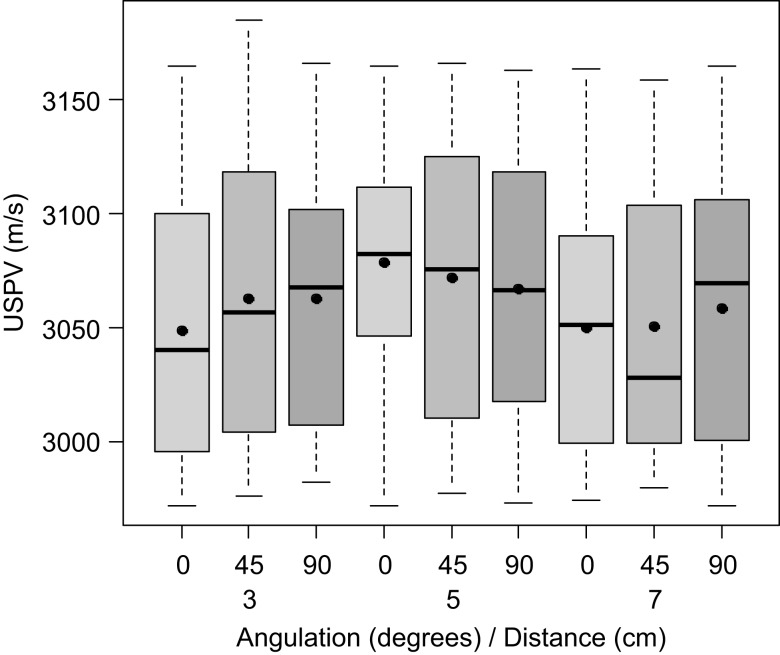
Ultrasound propagation velocity (m/s) comparison across the range of angulations of the transducers for each distance between them.

**Figure 7-:**
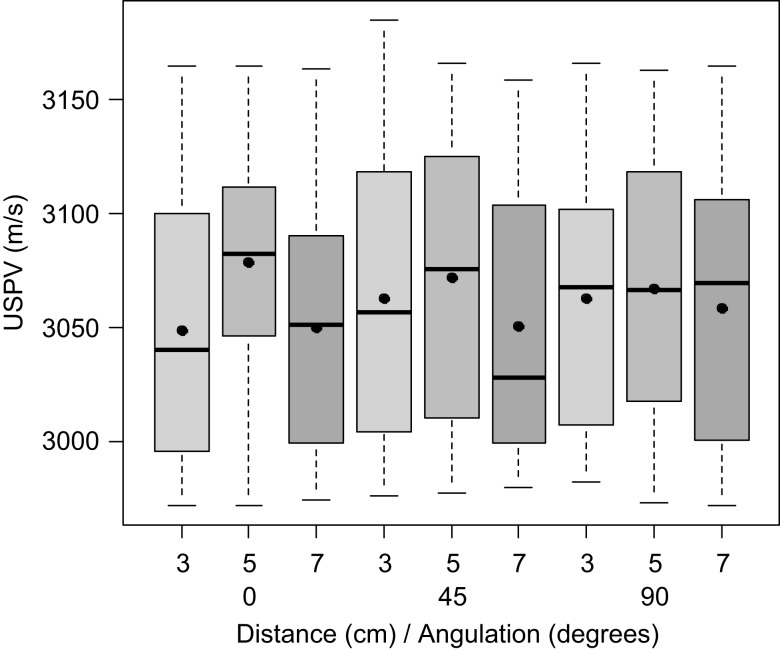
Ultrasound propagation velocity (m/s) comparison across the range of distances between the transducers for each angulation.

When the angulation of the transducers was fixed and the results obtained with different distances between them were compared, there was a significant difference between the 3 and 5 cm distances (*p*=0.0170) and between the 5 and 7 cm distances (*p*=0.0221) (both at an angulation of 0°). When the distance between the transducers was fixed and the results obtained with different angulations were compared, there was no significant difference between the set-ups. These findings are shown in Figures 6 and 7.

## DISCUSSION

Our subjects included equal numbers of men and women; all were adults with no medical conditions or a previous history of fracture of the clavicle. This enabled the data to be standardized and facilitated the statistical analysis. There was no significant difference in the measurements between genders or between sides, regardless of the distance between the transducers or their angulation. The observed absence of differences between the right and the left sides reveals that side dominance does not interfere with the measurements and that the contralateral clavicle could be used as a reference for measurements of the fractured clavicle in a clinical trial.

Altering the distance between the transducers and their angulation led to significant differences between the 3 and 5 cm distances and between the 5 and 7 cm distances (in both situations, the transducers were at an angle of 0° or parallel to the ground). The boxplot of these measurements shows that the means and medians in each comparison had no differences greater than 50 m/s; this value is of minor importance when considering the difference between minimum and maximum velocities (150 m/s) in all set-ups.

Although all transducer angulations produced similar results, the best angle was 45° because it best adapts to various physical types. A recent study using *ex vivo* bovine bone demonstrated that the distance between transducers does not interfere with the ultrasonic pulse velocity and that difficulty in capturing the signal can be caused by the power of the signal generator device 22. The greater the distance between the transducers is, the easier the adaptation to the clavicle is, considering a clinical situation in which the fracture site should remain in the center. The results obtained with this device and instruments in the present study, considering the anatomical characteristics of the clavicle, show that a clinical trial to monitor clavicle fracture healing using bone ultrasonometry would benefit from positioning the transducers at an angle of 45° and at 7 cm apart.

The main limitations of the study are the small sample size, despite the homogeneity of the results and the indiscriminate use of the technique in individuals of different ages and physical types, which are variables that were not addressed.

The quantitative use of ultrasound to investigate the physical properties of bones and fractures has been studied for approximately six decades. Although X-ray images are the gold standard in the diagnosis of most fractures, there is great interest in the possibility of using bone ultrasonometry as an ancillary technique for monitoring of fracture healing and the early detection of cases of delayed union and nonunion of fractures 2,. Quantitative ultrasonometry is less expensive, safer, and easier to use than other biomechanical methods of monitoring bone healing 5-7,26,. Moreover, this technique can be used outside the hospital environment.

In the case of long bones, ultrasonometry using axial transmission is the most widespread technique. Many studies have confirmed the feasibility of its use in the assessment of fracture union in simulated experimental models 25, and *ex vivo* experiments with animals 11,12,13,25,. There have been few *in vivo* studies on the subject, with some with animals 14,15,16 and others with humans. All of these studies focused on the tibia 18,1920^21^, probably because this subcutaneous long bone provides easy access for positioning of the transducers, because the soft tissues cause little interference and because a high number of cases is observed in traumatology. However, prior studies were all case series with the primary goal of describing a new method to monitor fracture healing, rather than performing a detailed statistical analysis.

Other bones, such as the ulna and the clavicle, exhibit an anatomy that facilitates the use of bone ultrasonometry because these bones are mostly subcutaneous. The possibility of applying and standardizing this technique in other locations opens up the field to new clinical trials, with the aim of including this technique in clinical practice as a method complementary to conventional radiography.

The use of bone ultrasonometry in the clavicle is feasible and the standardization of the technique proposed in the present study (transducers placed at an angle of 45° and at 7 cm apart) allows its future application in clinical trials to monitor the healing process of diaphyseal fractures of the clavicle.

## ACKNOWLEDGMENTS

São Paulo State Research Support Foundation (FAPESP – Fundação de Amparo à Pesquisa do Estado de São Paulo) – Grants 2007/56422-0 and 2008/55342-5.

## AUTHOR CONTRIBUTIONS

Mandarano-Filho LG was responsible for the experimental design, evaluation procedures, data interpretation and manuscript preparation. Bezuti MT was responsible for the evaluation procedures and data interpretation. Barbieri CH was responsible for the experimental design, financial support (FAPESP) and equipment provision.
